# Consequences of sex differences in Type I IFN responses for the regulation of antiviral immunity

**DOI:** 10.3389/fimmu.2022.986840

**Published:** 2022-09-16

**Authors:** Maria Pujantell, Marcus Altfeld

**Affiliations:** Institute of Immunology, University Medical Center Hamburg-Eppendorf, Hamburg, Germany

**Keywords:** sex-differences, type I IFN, antiviral immunity, inflammation, antigen-presenting cells (APC)

## Abstract

The immune system protects us from pathogens, such as viruses. Antiviral immune mechanisms aim to limit viral replication, and must maintain immunological homeostasis to avoid excessive inflammation and damage to the host. Sex differences in the manifestation and progression of immune-mediated disease point to sex-specific factors modulating antiviral immunity. The exact mechanisms regulating these immunological differences between females and males are still insufficiently understood. Females are known to display stronger Type I IFN responses and are less susceptible to viral infections compared to males, indicating that Type I IFN responses might contribute to the sexual dimorphisms observed in antiviral responses. Here, we review the impact of sex hormones and X chromosome-encoded genes on differences in Type I IFN responses between females and males; and discuss the consequences of sex differences in Type I IFN responses for the regulation of antiviral immune responses.

## Introduction

The immune system of females and males differs in the ability to respond to infections, resulting in differences in the incidence and manifestation of infectious diseases. Both gender- and sex-specific factors have been described to contribute to these differences between the sexes. In this article, we will focus on sex-specific factors determined by differences in sex hormones and genes encoded by the X chromosome. In particular, we will review the contribution of sex differences manifested in the Type I IFN pathway to the regulation of antiviral immunity.

## Type I IFNs

IFNs were first identified by Isaacs and Lindenmann in 1957 as antiviral molecules able to “interfere” with viral replication (1, 2). Later, IFNs were also described to modulate the function of the immune system, given their potent inflammatory and anti-proliferative properties. IFNs are soluble glycoproteins known as cytokines that are expressed by cells in response to stimuli such as viruses. More than thirty IFN genes on different chromosomes encode three major IFN types (IFN type-I, -II, -III) that are classified depending on their receptor usage and signaling pathways (3–6) (genenames.org). The diversity of IFNs observed in mammals and other vertebrates is thought to have arisen from a single ancestral *IFN-α* gene that has evolved into the different types of IFNs. This ancestral gene has been suggested to have duplicated early during vertebrate evolution, and that consequent retrotransposition events have led to the expansion of the IFN locus to produce the different IFN types, isotypes and subtypes in mammals (7–9). In humans, there are different isoforms of Type I IFNs (IFN-α, -β, -ϵ, -κ, -ω) encoded by chromosome 9, whereas the rest of Type II IFNs (IFN-γ) and Type III IFNs (IFN-λ1-4) only have a single subtype (7, 8, 10–12). Within the group of Type I IFNs, a total of 13 different *IFNα* genes have been described, but only one *IFNβ* gene. IFNα subtypes share 70-99% amino acid sequence identity between them, and only 35% similarity to IFNβ (13). This variation in gene and isoform numbers between Type I IFN genes points to an evolutionary benefit of having distinct IFNα subtypes (13); however the mechanisms by which different IFNα subtypes mediate differential immune responses are insufficiently understood, given the fact that all IFNα subtypes signal through the same IFNα receptors (IFNAR1/2). In this review, we will focus on the role of Type I IFNs in antiviral immune responses, as multiple studies have described sex differences in the induction of IFNα and IFNβ.

The signaling cascade used by Type I IFNs has been extensively studied to better understand the effects of IFNα and IFNβ during innate immune responses (14). Several pathways have been described to be induced by Type I IFNs, which can signal through both canonical and non-canonical signaling pathways. A canonical pathway refers to the generalized or conventional signaling pathway, whereas non-canonical pathways represent alternative signaling pathways that deviate from the most common or first-described canonical signaling pathway. However, both canonical and non-canonical pathways often converge intracellularly and lead to similar signaling. The canonical pathway of Type I IFNs (reviewed here (15)) signal through interferon-α receptor 1 (IFNAR1) and interferon-α receptor 2 (IFNAR2) subunits on the plasma membrane. These receptors signal intracellularly through janus kinase-signal transducer and activator of transcription (Jak/Stat) pathway and mitogen-activated protein kinase (MAPK) pathway (16). Phosphorylation of IFNAR, JAK1 and TYK2 recruits and phosphorylates STAT1 and STAT2 proteins, and forms a complex called IFN-stimulated gene factor 3 (ISGF3). ISGF3 then translocates to the nucleus, binds to IFN-response elements (ISRE) on promoters, and induces expression of interferon-stimulated genes (ISGs). The array of ISGs induced will subsequently determine the effects of Type I IFNs on inflammation and immunity. It is known that different subtypes of IFNα and IFNβ induce different ISG patterns in different cells (17), with distinct downstream consequences for activation of immune effector cells (18, 19). In contrary, non-canonical Type I IFN signaling pathways also involve binding of IFNs to the IFNARs, and induce activation through serine phosphorylation of JAK1/TYRK2, instead of tyrosine phosphorylation (20). The non-canonical pathways furthermore use different intracellular mediators to induce Type I IFNs and also result in altered ISG expression (reviewed in (21)). To date, three different non-canonical signaling pathways have been described: MAPK-, PI3’K/mTOR- and CDKs-pathways. The MAPK pathway involves MAPK protein mediators such as Jnk, ERK or p38. Protein p38 is known to phosphorylate STAT3 and increase expression of PD-L1, IL-6 and IL-2 in mature dendritic cells, possibly contributing to inflammation (22). Although the MAPK pathway is considered non-canonical, it can thereby complement the function of canonical JAK-STAT pathway. The PI3’K/mTOR non-canonical pathway has been linked to translation of ISG mRNA, and is involved in IFN-dependent gene transcription through interferon-stimulated response elements (7, 23–25). Lastly, cyclin-dependent kinases (CDKs), such as CDK1, CDK2 and CDK4, can affect translation of IFNβ (26) and CDK8 is known to regulate STAT transcription activation (27), thereby affecting Type I IFN production. Taken together, Type I IFNs use canonical and non-canonical signaling pathways that can control activation and regulation of IFNs and ISG expression, ultimately regulating transcription, translation and function of Type I IFN responses.

## Sex differences in Type I IFN responses

IFNs are produced by different innate immune cells, in particular antigen presenting cells (APCs) such as macrophages, monocytes and dendritic cells. Plasmocytoid dendritic cells (pDCs) have the ability to produce high amounts of Type I IFNs, with one pDC able to secrete between 1 to 2 IU of IFNs in response to viral stimuli, which is up to 100 times more than described for other immune cells (28, 29). pDCs can produce all Type I, II, III IFNs, although type II IFNs have only been detected in low amounts (30), and also express the receptors binding these IFNs, thereby serving as central modulators of immune responses. Importantly, several studies have demonstrated that the Type I IFN response of pDCs is stronger in females compared to males (31–36). Sex differences have been described in the ability of pDCs to produce IFNα after toll-like receptor 7- (TLR7) stimulation, while data on the effect of sex on toll-like receptor 9- (TLR9) mediated response is less clear. Initial studies showed that PBMCs of females produced higher amount of IFNα in response to TLR7-stimulation compared to males, and that this variance was due to sex differences in IFNα production by pDCs (31, 32). This sex difference was not explained by different frequencies of pDCs between females and males or direct *in-vitro* effects of estrogen on pDCs (31), but was linked to a higher frequency of pDCs that produced IFNα following TLR7-stimulation (32, 33, 35). Subsequent studies demonstrated a contribution of both sex-chromosomal and sex-hormonal factors that enhance the ability of female pDCs to produce higher IFNα in response to TLR7-stimulation (34). Altogether, these data suggest that sex differences in Type I IFN responses by pDCs can contribute to better immune responses to viral infections and vaccinations in females. Below we will review the role of sex hormones and genes encoded by the X chromosome on sex-specific differences in Type I IFN responses.

### Effects of sex hormones on antiviral immune responses

Receptors for sex hormones, including estrogen receptors (ER) and androgen receptors (AR), are expressed by most immune cells, and sex hormones can therefore affect the function of many immune cells, including pDCs and their ability to produce Type I IFNs. Females mostly produce estrogen and progesterone, while testosterone is the principal sex hormone in males; furthermore, sex hormone levels also depend on the age of the individual. Given the differences in estrogen and testosterone levels between the sexes, it is important to note that males and females appear to express similar levels of hormone receptors in immune cells, based on data from bulk mRNA-seq data (dice-database.org). In general, the majority of studies have shown that estrogen has an immune-stimulatory effect whereas testosterone displays an immune-suppressive effect.

**Figure 1:**
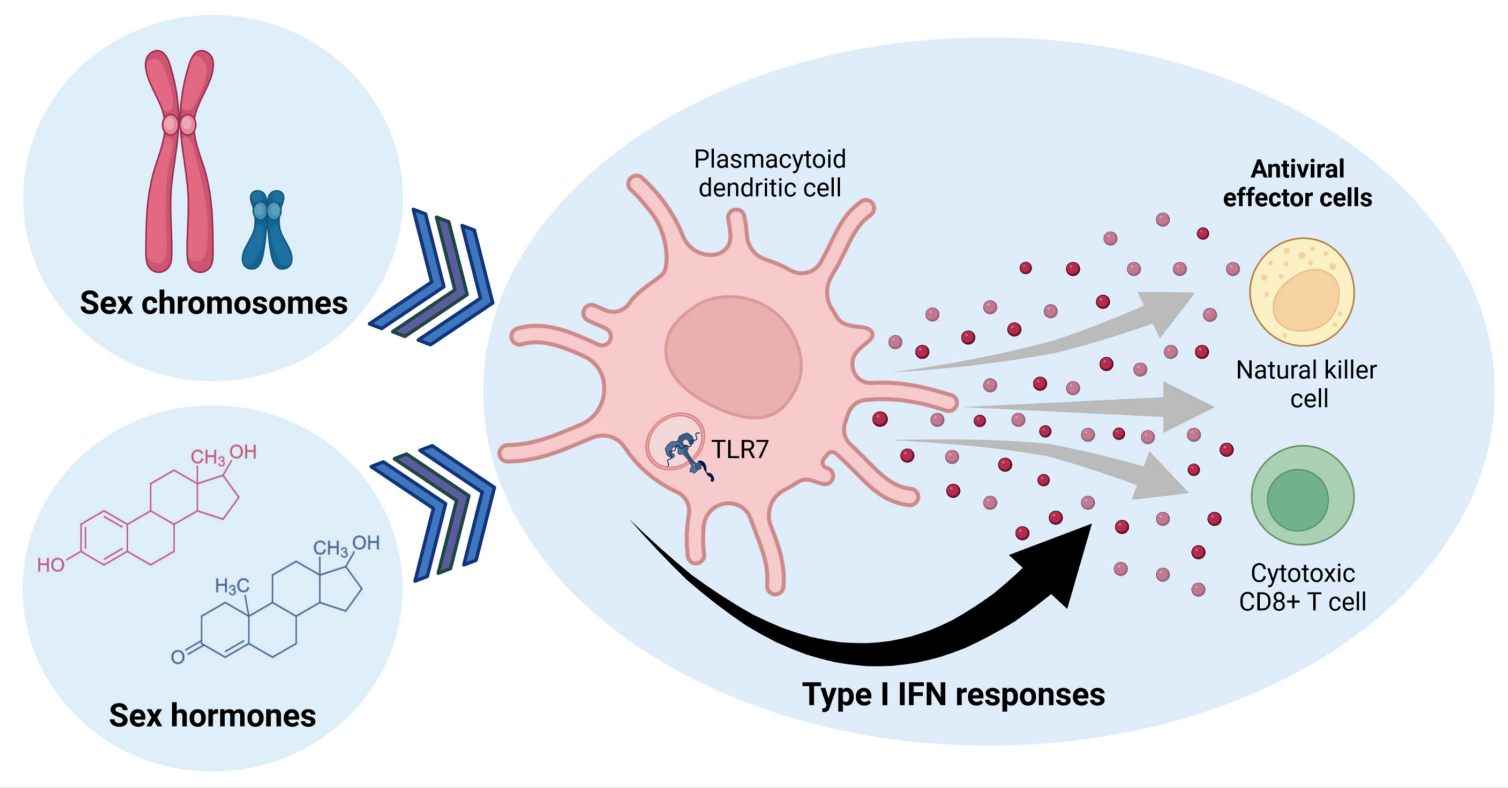
Sex determining factors influence Type I IFN responses in plasmocytoid dendritic cells, which can regulate antiviral effector cell functions of natural killer and cytotoxic CD8^+^ T cells. Sex hormones and genes encoded by sex chromosomes can alter TLR7-signaling in pDCs, modulating expression of Type I IFNs that affect other immune cells. Figure created with BioRender.com.

There are two estrogen receptors, ERα and ERβ, expressed on mature immune cells, but also their hematopoietic precursors (37). The ERs serve as ligand-dependent transcription factors, enabling ERs to modulate immune function by directly regulating gene transcription, including NF-κB or IRF5 transcription (35, 38–40). Particularly within pDC and B cells, levels of *ESR2* mRNA have been reported to be higher expressed compared to other immune cell types (34, 41), suggesting that estrogen has the ability to impact Type I IFN responses in pDCs. One study by Seillet et al. showed that estrogen treatment in postmenopausal females enhances the production of IFNα and TNFα by pDCs after TLR7- and TLR9-stimulation (33). Moreover, ovariectomized mice exhibited decreased production of IFNα and TNFα by pDCs after TLR7-stimulation (33), which was shown to depend on the expression of ER in the hemapoietic compartment in mice (33). Overall, estradiol has been reported to affect pDC differentiation, induce transcription factors IRF4 and IRF5, and enhance the frequency of IFNα- and TNFα-producing pDCs after TLR-stimulation (33, 35, 39, 40). In murine pDCs, ablation of ER decreased *Irf5* mRNA levels and reduced the percentage of IFNα/IFNβ-producing pDCs (35). Expression levels of transcription factor IRF5 directly correlated to the percentage of IFNα-producing pDCs after TLR7/8-stimulation, as well as other inflammatory cytokines (35). In a mouse model of Flt3L DC differentiation, ERα signaling potentiated the ability of pDC to produce pro-inflammatory cytokines after TLR-stimulation, contributing to sex differences in female pDCs (42). Additionally, using mouse models with pDCs-specific ER KO, it was shown that ER-signaling positively regulated TLR7-induced production of Type I IFNs, further supporting the important effects of estrogens in regulating IFN production after TLR7- and TLR9-stimulation (33, 35, 43). Taken together, these data suggest that estrogens have the potential to modulate immune-pathways affecting Type I IFN responses of pDCs, and might thereby contribute to the observed enhanced production of IFNα by pDCs following TLR7-stimulation in females (**Figure 1**).

On the other hand, there is only one gene encoding for the androgen receptor (*AR)*, which can form different protein isoforms, the full-length AR (AR-FL) and several AR splice variants (referred to AR-Vs) that have been associated to androgen-independent growth in cancer (44). Effects of androgens have been described for the development, activation and functional modulation of pDCs. Of note, males transitioning from pre- to post-puberty showed an increase in IFNα-producing pDCs after TLR-stimulation. Important to note, females still maintained higher percentages of IFNα-producing pDCs than males (45). Moreover, treatment of pDCs from females with DHT, a form of testosterone, has been shown to decrease IFNα-production in response to TLR7-stimulation *in-vitro (* 46), highlighting how testosterone treatment can indeed dampen IFNα responses in female pDCs. Overall, these data demonstrate an important role of sex hormones in regulating IFN responses (**Figure 1**), but further research is required to better understand the precise involvement of sex hormones in the regulation of expression, translation and function of specific genes that modulate Type I IFN responses in humans, in order to identify novel targets for immunotherapeutic interventions.

### Effects of genes encoded by the X chromosome on antiviral immune responses

The X chromosome encodes for about 1200 genes (47), and thereby more genes than the Y chromosome. Genes on the X chromosome comprise many immune-regulatory molecules, including TLR7, TLR8, FoxP3, IL-2RG, IL3RA, IRAK1, BTK, NKAP, WAS, CYBB, AR, CSF2RA, EPAG, GATA1, CD99, CD40L, HDAC6, HDAC8 (48–50). Females have two X chromosomes; and in mammals, female cells undergo random inactivation of one of the two X chromosomes. X chromosomal inactivation (XCI) is subsequently inherited by daughter cells through somatic cell division. This process is thought to be partly controlled by XIST, the X inactive transcription factor, which keeps the inactive X chromosome genes transcriptionally silenced in females (51). Furthermore, additional factors have been suggested to contribute to the maintenance of XCI besides XIST, including long noncoding RNAs, chromatin modifications and nuclear organization (52–54). However, the inactivation of the second X chromosome is not complete, and recent data have shown that genes located on the second X chromosome can escape from XCI in a subset of cells, including immune cells (55–58). In particular, in some lymphocyte populations, such as B and T cells, XCI regulatory elements have been described to be lost after cell activation, facilitating escape from XCI in activated immune cells (52, 54, 59). Furthermore, certain regions within the X chromosome are more prone to escape from XCI than others, such as the short arm of the X chromosome, also known as Xp (55). Escape from XCI can therefore result in higher expression levels of X-chromosomal encoded genes and proteins in subsets of immune cells in females. Patterns of XCI have been described as both random and/or tissue-specific (55), emphasizing the complexity and variability of this process that can result in higher transcriptional, translational and functional heterogeneity in immune cells derived from females compared to males. Importantly, several of the molecules that can impact Type I IFN responses through either direct or related pathways are encoded by the X chromosome, including TLR7, TLR8, IL2RG, NKRF, DDX3X, UTX, EPAG, FOXP3 and IKKγ (48, 60). Moreover, 10% of all miRNAs are also encoded by the X chromosome and could possibly regulate transcription of other key autosomal genes (61). These genes are linked to modulation and activation of immune responses, including pro-inflammatory responses that can trigger and affect IFN signaling pathways. Escape from XCI of these miRNAs is less well understood, but might contribute to heterogeneity of Type I IFN signaling and antiviral immune responses in females. Below we focus more specifically on escape from XCI of *TLR7* gene and its consequences for sex differences in Type I IFN responses, given recently described functional consequences of *TLR7* escape from XCI.

Escape of *TLR7* from XCI has been reported in several immune cells, such as B cells, monocytes and pDCs (57). Moreover, males with Klinefelter syndrome (47,XXY) also display *TLR7* XCI escape due to the presence of two X chromosomes in pDCs, monocytes and B cells (57). Quantification on a single cell level of *TLR7* escape from XCI in B cells and pDCs showed enhanced *TLR7* mRNA (56, 57) and TLR7 protein expression in pDCs (56). *TLR7* escape from XCI has been furthermore linked to enhanced production of Type I IFNs and enhanced innate antiviral immune responses after TLR7-stimulation (56, 57) (**Figure 1**). Additionally, pDCs with *TLR7* escape from XCI also exhibited increased transcription of different *IFNα* subtypes and *IFNβ* mRNAs (56). Taken together, the case of *TLR7* provides an example on how escape of a gene from the inactivation of the second X chromosome can significantly increase the production of IFNα in female pDCs, and thereby potentially enhance antiviral immunity but also increase the risk for autoimmune diseases. More in depth studies investigating the biological consequences of other genes escaping XCI for sex differences in immune responses are therefore warranted.

Importantly, the effects of sex hormones and sex chromosomes cannot be investigated in isolation, as a number of recent studies have indicated important interactions between these two factors in regulating Type I IFN responses in pDCs. One study analyzed a unique group of young volunteers, which included females, males, transgender males, transgender females and volunteers with Turner syndrome (XO) to understand the contribution of X chromosome numbers and serum sex hormones to the ability of pDCs to produce Type I IFNs after TLR7-stimulation (45). The study described a positive association between the percentage of IFNα-producing pDCs after TLR7-stimulation and testosterone levels in the presence of one X chromosome, but a negative association in the presence of two X chromosomes (45). This data suggests a bi-directional association depending on the number of X chromosomes, and underlines the complex interaction between X chromosome gene dosage and hormone regulatory effects. Moreover, it highlights the importance in studying unique cases with chromosomal number variations to understand the individual effect of sex chromosomes and X chromosome-encoded genes. Interestingly, hormones can affect gene expression. It is now well-established that both gene expression and DNA methylation patterns of immune cells can be modulated by sex hormones (62). Methylation patterns appear early during embryonic development before the production of sex hormones (63), and are later exacerbated and modulated by the effects of hormones, inducing putative sexual dimorphism effects (63). Unfortunately, since DNA methylation plays a role during mammalian XCI, most methylation studies disregarded methylation patterns in the X chromosome in order to obtain reliable results, as gender was often not considered a modulating factor. In one study, several autosomal genes were identified to be differentially methylated in a sex-specific manner (62). These hypermethylated genes found in females were linked to immune molecules such as CD3, lymphocyte-specific protein 1(LSP1), IgM, BCL11B, TNFRSF4, and the NFKB complex, and associated to cell-mediated immune response pathways. Additionally, using network analysis, these autosomal sex-specific differentially methylated genes were linked to estrogen receptor ER in females (62). These data suggests a pivotal connection between estrogen and methylation patters differentially regulating sex-specific immune responses (62). Therefore, studying the interdependence between gene dosage in sex chromosomes, hormonal immune regulation and DNA methylation patterns will reveal important additional layers of immune regulation, and will be crucial to understand sex-specific differences in immune responses.

## Antiviral and inflammatory effects of Type I IFNs – implication for sex differences in manifestations of viral infections

Type I IFN responses represent a critical early component of the antiviral immune response, but can also contribute to immunopathology when dysregulated by inducing excessive inflammation and promoting viral replication. Indeed, several studies have shown that most viruses dysregulate key molecules upstream and/or downstream of the IFN-induction cascades to prevent immune detection by regulating transcription, translation, RNA processing, trafficking and degradation of host proteins to their advantage [review in (64)].

Immune cells recognize pathogens though the identification of pathogen-associated molecular patterns (PAMPS), which are conserved small motives unique to pathogens (65). At the same time, damage-associated molecular patterns (DAMPS), which are molecules produced by infected or damaged cells, are also recognized by immune cells. In innate immune cells, pattern recognition receptors (PRRs) detect the presence of intracellular PAMPS and DAMPS and trigger signal cascades. Different classes of PRRs exist and detect diverse structures of PAMPS. Toll like receptors (TLRs) are transmembrane sensors found in the endosomes and cell membrane, and some TLRs have the ability to directly induce cytokines and IFNs after pathogen detection (65), as already described for TLR7. Transmembrane c-type lectin receptors (CLRs) detect carbohydrates in a calcium-dependent manner. Nucleotide-binding oligomerization domain (NOD)-like receptors (NLRs) are intracellular sensors that identify bacterial products, for instance peptidoglycans. Retinoic acid-inducing gene I (RIG-I)-like receptors (RLRs) bind to cytosolic viral ssRNA or dsRNA, while other cytosolic DNA-sensors such as cGAS bind to dsDNA and signal downstream after detecting viral nucleic acids. To date, most studies investigating the mechanisms underlying sex differences in innate immune responses have focused on differences in TLR7 expression, as this receptor is encoded by the X chromosome and has been implicated in sex differences in sensing pathogens by several studies, as described above. However, enhanced expression of other PRRs in females might also contribute to sex differences in the response to viral infections. For instance, RIG-I is an IFN-stimulated gene that, due to overall enhanced Type I IFN responses in females, might increase its expression faster in female than male cells. Indeed, RIG-I signaling has been shown to be targeted in viral infections, suggesting a viral mechanisms to avoid detection during hepatitis C virus (HCV) infection (66). HCV also disrupts STING signaling complexes, cleaves MAVS and TRIF proteins, all of them limiting IFN induction (64). Other viruses, such as dengue virus (DENV) or sendai virus (SeV), antagonize STAT-signaling proteins by inhibiting STAT protein phosphorylation and their nuclear translocation, thereby weakening ISG induction and affecting the IFN response at different levels (64). To sum up, most viruses have evolved different mechanisms to weaken IFN signaling for their benefit, and these effects on Type I IFN responses can differ between the sexes. Females tend to have lower viral loads during primary infections with HIV (67), HCV (68) or respiratory virus (69) compared to males, while stronger immune-mediated pathology during chronic infection can be observed in females [reviewed in (70)].

Moreover, downstream signaling of PRRs might also be affected by sex. After PRRs activation, protein kinases phosphorylate and activate different signaling mediators, such as myeloid differentiation primary response 88 (MYD88) or mitogen-activated protein kinases (MAPKs). These mediators lead to induction of transcription factors, including nuclear factor-kB (NFKB) or IFN-regulatory factors (IRFs). Different components of these intracellular molecules are encoded by the X-chromosome, which include *NKRF* or *IKBKG (* 48, 60). Escape from XCI of these genes in some cells can therefore promote signal transduction in females. At the same time, higher expression of IFNs in female cells enhance IFN-regulated transcription factors, such as IRFs. Furthermore, transcription factors have been reported to display sex-bias, and target different genes depending on the specific human tissue being analyzed (71). A recent study performed sex differential expression analysis and gene regulatory network analysis using a large dataset of healthy adults from The Genotype-Tissue Expression (GTEx) project (71). The authors used two network modeling methods, PANDA and LIONESS, to infer sample-specific gene regulatory networks from different healthy human tissue types (71), and found that most sex differences were involved in the regulation of transcription rather than differences in actual mRNA expression in the tissue. These results point at the importance to identify central transcription regulators, and the possible limitations of only considering RNA expression. Overall, these data suggests that tissue-specific regulation of Type I IFN responses might differ between sexes. Further studies are therefore required to detangle tissue-specific regulatory mechanisms that influence sex differences in antiviral immune responses in diverse tissues.

## Impact of Type I IFNs on antiviral effector cells, and implications for sex differences in antiviral immunity

In addition to the direct antiviral effects of Type I IFNs described above, Type I IFNs also play a central role in induction and modulation of antiviral effector cells. Therefore, sex differences in Type I IFN responses can have important implications for the subsequent effector cell-mediated control of viral infections. Although many immune cell subsets, including macrophages, neutrophils and dendritic cells, are affected by Type I IFNs and can contribute to an effective antiviral immune response, we will focus here on the effect of Type I IFNs on the two principal antiviral cytotoxic effector cells, NK cells and virus-specific CD8^+^ T cells (**Figure 1**).

### Type I IFN-mediated effects on NK cells, and implication for sex differences in antiviral immune responses

NK cells are cytotoxic innate lymphocytes that play an important role in the clearance of tumor or virus-infected cells. Antiviral NK cells express multiple receptors that enable the recognition and killing of infected cells (reviewed by (72) and (73)). Activated NK cells produce IFNγ (Type II IFNs) and TNF-α, and cytotoxic molecules such as perforin and granzymes that induce the killing of target cells. Several studies have shown that the antiviral effector function of NK cells is modulated by Type I IFN responses (53, 74), suggesting that sex differences in Type I IFN responses might lead to differences in NK cell function between the sexes.

Type I IFNs are important for many NK cell processes, such as maturation and development. Mice lacking IFNAR1 or IFNAR2 showed reduced NK cell maturation at early stages without altered total NK cell numbers in spleen and blood (75–77), suggesting an essential role for Type I IFNs in the development of functional NK cells. Human inborn errors in IFNAR2 furthermore resulted in a dysregulation of NK cell function after IFNα stimulation (78). NK cells from these individuals did not exhibit an increase in degranulation and cytotoxic function after IFNα-stimulation due to a lack of functional IFNAR and consequent signaling (78). These data are in line with earlier studies reporting that NK cells increase their cytotoxicity and effector mechanisms, such as Fas-L and perforin expression, after IFNα stimulation (74). Additionally, transcription factors critical for IFN signaling, such as IRF3 or IRF5, are essential for efficient NK cell activation (79), suggesting a direct link between NK cell activation through Type I IFNs and enhanced NK cell cytotoxicity against viral infections. Type I IFNs activate STAT molecules, and depending on the accessibility and abundance of STAT proteins can induce unique signaling pathways triggering antiviral responses (reviewed here (80)). Many studies have described the involvement of JAK-STAT pathway during development and maturation of NK cells, and how these can modulate cytokine production and killing efficiency of NK cells (81–83), suggesting that different components of Type I IFN response can all modulate antiviral responses in NK cells.

Lastly, hormones can also affect IFNγ production of murine NK cells. Testosterone treatment of female mice reduced IFNγ production by NK cells, whereas orchiectomized male mice display increase IFNγ production by NK cells (84). Additionally, the percentage of human NK cell subpopulations in peripheral blood were found to be modulated by both sex and age (85–87). Some studies of NK cells have shown differences in cytotoxicity and cytokine production depending on age and sex (84, 88, 89), although other studies have reported no differences (90, 91). These discrepancies might be explained by differences in subjects age and hormonal levels, as NK cells in females have been reported to be altered during the menstrual cycle (91, 92) and by oral contraceptives (89). In one study, estrogen levels did not show any correlation to NK cell function (93), while progesterone has been suggested to induce apoptosis of NK cells (92) leading to reduced NK cell cytotoxicity. Overall, the results from these studies suggest that sex-specific differences in NK cell function can result from different mechanisms, including differences in Type I IFN signaling and direct, as well as indirect effects of sex hormones on NK cell function (**Figure 1**). Future studies are required to determine the relative contribution of these different factors, and to differentiate between sex-specific differences in NK cell-intrinsic pathways and those mediated by NK cell-extrinsic factors, such as differences in IFNα-levels between the sexes. Furthermore, the consequences of these sex differences in NK cell responses for both antiviral immunity and immune-pathogenesis need to be better understood.

### Type I IFN-mediated effects on CD8^+^ T cells, and implication for sex differences in antiviral immune responses

CD8^+^ T cells are a central part of the adaptive immune response against viruses (94–97). Cytotoxic CD8^+^ T cells can kill virus-infected cells through the release of cytokines and cytotoxic molecules, similar to NK cells, and generate long-term memory CD8^+^ T cells. Type I IFNs are important for survival, cytokine production, clonal expansion, and memory formations of CD8^+^ T cells during viral infections (98, 99) (**Figure 1**) through both direct and indirect mechanisms (100). Sex-specific differences in Type I IFN responses might therefore translate into sex difference in antiviral CD8^+^ T cell functions.

Several studies have suggested sex-specific differences in CD8+ T cell function. Human female CD8^+^ T cells have been reported to be more prone to activation and proliferation compared to males after PHA-stimulation of PBMCs (87), with female T cells displaying stronger upregulation of antiviral and pro-inflammatory genes after stimulation (87). Importantly, half of the overexpressed genes in females had estrogen responsive elements in their promoter regions (87), suggesting a direct regulatory role of sex hormones on CD8^+^ T cells. Sex differences have furthermore suggested to shape virus-specific T cell subsets in human lymphoid and mucosal tissues (101). Females were reported to maintain higher frequencies of CMV-specific CD8^+^ T cells in the lungs than males, while such sex-specific differences were not reported for influenza-specific CD8^+^ T cells, suggesting sex as a factor driving virus-specific T cell responses in some infections (101). Interestingly, a sex-bias was present in the subset composition of CMV-specific CD8^+^ T cells, suggesting that both differentiation and maintenance of virus-specific CD8^+^ T cell can be regulated by sex (101). Furthermore, *in-vitro* treatment of virus-specific mice CD8^+^ T cells with different IFNα subtypes has been reported to enhance their function, measured by enhanced IFNγ, IL-2 and TNFα-production, but was at the same time also able to suppress CD8^+^ T cell proliferation (99). These opposing effects of Type I IFNs on CD8^+^ T cell have been suggested to induce a more balanced T cell response by keeping proliferation of highly cytotoxic CD8^+^ T cells under control. The precise mechanisms by which Type I IFNs regulate CD8^+^ T cell function are not fully understood, and different mechanisms have been proposed. In mice, IFNAR-deficient virus-specific T cells are eliminated by NK cells after LCMV infection in contrast to IFNAR-expressing virus-specific T cells, an effect that is rescued after NK cell depletion (102, 103). These data suggest that Type I IFN signaling might not directly affect CD8^+^ T cell effector functions but indirectly regulate CD8^+^ T cell immunity through NK cells. IFNα treatment in CD8^+^ T cells enhanced the expression of MHC class I and Qa-1b molecules, which serve as ligands for inhibitory NK cell receptors, in an IFN concentration-dependent manner after co-incubation with αCD3 (103). Furthermore, IFNAR-KO T cells have also been described to upregulate the expression of ligands for the activating natural cytotoxicity receptor 1 (NCR1) upon infections, rendering IFNAR-KO CD8^+^ T cells as targets for NCR1-mediated NK cell killing (102, 103). Type I IFN signaling appears to also be important for memory formation of T cells, as IFNAR-KO T cells exhibited enhanced formation of memory precursor effector cells (MPECs) and reduced formation of short-lived effector cells (SLECs) in mice (102). Data linking sex-specific differences in Type I IFN production to sex-specific T cell responses are very limited. One study described enhanced and more differentiated CD8^+^ T cell responses after infections in female compared to male mice; however, this effect was not explained by Type I IFN signaling, but enhanced sensitivity to IL-12 in female compared to male CD8^+^ T cells (104). Exposure to both, IL-12 or Type I IFNs, can enhance the expression of CD25, a high affinity IL-2 receptor, on CD8^+^ T cells (105), pointing to potential sex differences in the regulation of CD8^+^ T cell responses by direct and indirect mechanisms. Taken together, Type I IFN signaling has a critical role in regulating virus-specific CD8^+^ T cell functions, and sex-specific differences in Type I IFN production following viral infections might thereby impact antiviral CD8^+^ T cell responses. However, sex hormones can also directly regulate T cell functions, with consequences for antiviral immunity.

## Concluding remarks

Type I IFNs are central players in antiviral immunity due to their direct antiviral properties and their ability to regulate the development of an antiviral immune response. Biological sex is an important modulator of Type I IFN responses, both through sex-hormonal and sex-chromosomal factors, resulting in stronger Type I IFN responses by pDCs in females, with consequences for the clinical manifestations of viral infections. Future studies will need to identify the precise mechanisms resulting in sex-specific differences in Type I IFN responses during viral infections, in order to develop antiviral strategies that account for sex as an important biological variable.

## Author contributions

MP reviewed the literature. MP and MA wrote and reviewed the manuscript. All authors contributed to the article and approved the submitted version.

## Funding

This work was supported by the Deutsche Forschungsgemeinschaft (DFG) Research Unit 5068-Sex differences in immunity. Open Access funding enabled and organized by Projekt DEAL.

## Conflict of interest

The authors declare that the research was conducted in the absence of any commercial or financial relationships that could be construed as a potential conflict of interest.

## Publisher’s note

All claims expressed in this article are solely those of the authors and do not necessarily represent those of their affiliated organizations, or those of the publisher, the editors and the reviewers. Any product that may be evaluated in this article, or claim that may be made by its manufacturer, is not guaranteed or endorsed by the publisher.
